# Efficiency of the new reciprocating and rotary systems with or without ultrasonics in removing root-canals filling with calcium silicate-based sealer (MTA)

**DOI:** 10.1186/s12903-022-02684-3

**Published:** 2023-01-03

**Authors:** Ahmad A. Madarati, Aya M. N. Sammani, Ahmad A. Alnazzawi, Ali Alrahlah

**Affiliations:** 1 Restorative Dental Sciences Department, College of Dentistry, Taibah University, Madina, Kingdom of Saudi Arabia; 2 Department of Substitutive Dental Sciences, College of Dentistry, Taibah University, Madina, Kingdom of Saudi Arabia; 3 Restorative Dental Sciences Department, Engineer Abdullah Bugshan Research Chair for Dental and Oral Rehabilitation, College of Dentistry, King Saud University, Riyadh, Kingdom of Saudi Arabia; 4 Riyadh, 13311 Kingdom of Saudi Arabia

**Keywords:** Endodontics, Micro-ct, MTA, PUI, Reciprocating, Rotary, Retreatment, Sealer

## Abstract

**Background:**

To compare the efficiency of endodontic *rotary* and *reciprocating* systems in removing calcium silicate-sealer based fillings and to investigate the impact of passive ultrasonic irrigation (PUI) on their efficiency.

**Materials and methods:**

160 root-canals were instrumented, filled with gutta-percha and calcium silicate based-sealer and divided into 10 equal groups. Five groups in which the *reciprocating* systems (*WaveOne-Gold*, *Reciproc-Blue* and *R-Motion)* and *rotary* systems (*Fanta-AF-One* and *Tango-Endo)* were used to remove root-canals’ fillings. In the other five groups the fillings were removed by the same systems then additionally with PUI. The times to complete retreatments procedures were recorded. Micro-computed tomography’s analysis of the root-canals fillings’ volume before and after retreatments was used to determine the remaining filling materials (RFMs) volumes.

**Results:**

The RFMs after using *rotary* systems (10.1%) was greater than after using *reciprocating* systems (3.8%) (*P* < *0.001*). The RFMs after using *WOG* (2%) and *RB* systems (2.6%) were less than those in the *RM (*6.8%), *TE* (9.5%) and *FAFO* (10.7%) systems *[P* < *0.05].* The times required to remove the filling materials using the *TE (*3.7 min), *FAFO* (4.1 min) and *RM* (4.1 min) systems were shorter than those required by the *RB* (5.4 min) and *WOG* (4.9 min) systems *[P* < *0.05]*. Using PUI resulted in less RFMs (1.44%) when compared to using only *rotary* or *reciprocating* systems (6.27%) *[P* < *0.001]*.

**Conclusions:**

Endodontic *reciprocation* systems were more effective, but needed longer times than *rotary* systems in removing calcium silicate based- sealers fillings. The PUI significantly improved removal of the root-canals’ filling materials.

*Clinical relevance*: Reciprocating systems and PUI are recommended whenever root-canals retreatment is considered regardless of using calcium silicate-based sealers.

## Introduction

Long-term success of root-canals treatments (RCTs) is not always warranted, as it depends on correct clinical procedures and systematic factors [1]. If an RCT fails, a non-surgical root-canal retreatment is an option to uncover the areas of the root-canal system that harbor microorganisms or infected remnants. Therefore, retreatability has been one of the main properties of an ideal root-canals filling materials to facilitate retreatments [2]. Gutta-percha has been the gold standard core root-canals’ filling material with long history of clinical success. However, there has been no agreement on the superiority of one sealer over another, hence this area is still attracting intensive *in-vitro* and *in-vivo* research. Calcium-silicate-containing sealers (CSCSs) have been introduced and remarkably investigated. Though *in-vitro* studies have shown their excellent properties, clinical long-term success is yet to be established. Recent studies, with relatively short follow-ups, showed promising outcomes [3, 4]. CSCSs are hard upon setting, create hydroxyapatite crystals upon their interface with dentine, and can penetrate the dentinal tubules [5]. Consequently, their retreatability and regaining patency to the root-canals full length (to properly disinfect them) is one main concern, despite their increased adoption. The literature shows conflicting results in this regard. Two studies found inferior removal of CSCSs-based fillings compared to those of AH-26 [6] or AH-Plus sealers-based fillings [7]. Hess et al, [8] also, found more remaining filling materials (RFMs) within CSCSs groups and patency was not re-established in 20% of samples filled with CSCSs. By contrast, Donnermeyer et al [9] found greater RFMs in the AH-Plus groups compared to the CSCSs groups. Many studies did not find significant differences between CSCSs and the AH-plus sealer [5, 10–12]. These studies also showed conflicting results regarding the time required to perform retreatment procedures. However, Sfeir et al, [13] stressed on the need for further studies that better simulate the real clinical conditions of retreatment cases to avoid the methodological bias observed in many studies. Nevertheless, studies investigating CSCSs retreatability confirmed that no methods or techniques can remove old root-canals filling materials completely. Therefore, there has been always need for more effective instruments and supplementary methods to maximiz removal of root-canals fillings materials. Some studies have reported improved removal of filling materials following supplementary agitation of irrigants by laser, sonic or ultrasonic vibrations [14–17]. Also, reciprocating systems showed promising results in this regard [9, 18–21]. The *R-Motion* (*RM*) system (FKG Dentaire, Switzerland) is a new nickel titanium single-file system that works in a reciprocating motion. The only study about it showed that this file had a similar shaping ability to that of *Reciproc-Blue* (*RB*), *HyFlex-CM* and *XP shaper* systems [22]. The *Tango-Endo* (*TE*) (Essential Dental Systems, NJ, USA) and *Fanta-AF-One* (*FAFO*) (Fanta Dental, China) are new NiTi rotary systems with a completely flat design along one side of the file’s active part. In a pilot experiment, both systems, when rotated continuously, were able to remove root-canals filling materials. To the best of our knowledge, there have been no reports on the retreatment ability of *RM, TE, or FAFO* systems. Therefore, authors believed that this knowledge’s gap needs to be addressed.

Different methods and techniques have been adopted to measure the RFMs after retreatments. Three-dimensional (3-D) measurement using micro-computed tomography (micro-CT), is accurate and more accepted among researchers [23]. Therefore, the current study aimed at (a) comparing the effectiveness of different *rotary* and *reciprocating* systems in removing filling materials with an MTA-type sealer (as an example of calcium silicate sealers) and (b) investigating the impact of passive ultrasonic irrigation (PUI) as a supplementary method on removal of the RFMs by micro-CT analysis. The two tested null hypotheses were:

I. There would be no significant differences among the *rotary* and reciprocating systems in removing filling materials with an MTA-type sealer.
II. There would be no significant differences in RFMs between groups with passive ultrasonic irrigation (PUI) and those without PUI.

## Methods

### Ethical approval and samples preparation

This study was approved by the National Science Technology and Innovation Plan’s ethical committee (KSA) (Ref: 13-BIO57-05) and was performed in accordance with the Declaration of Helsinki. The National Science Technology and Innovation Plan’s ethical committee waivered the need for informed consent. This is because the upper premolars (with two separated roots) used in this study were collected from pools of teeth that had been extracted before the study for periodontal and orthodontic reasons, but not for the purpose of this study. In addition, this manuscript was written according to the Preferred Reporting Items for Laboratory studies in Endodontology (PRILE) 2021 guidelines [24]. The premolars with previous RCTs, coronal restorations, caries, fractures, internal or external resorptions, had canals curvature angles greater than 30 degrees (by Schneider method using bucco-lingual and mesiodistal projections radiographs) [25], or had extra canals were excluded. This study was the second part of a research project that had been conducted earlier, in which the first study’s sample size calculation (manuscript has been accepted recently) suggested 15 samples in each group considering 90% power calculation to detect differences when standard deviation is 3.950. Nevertheless, the sample size in the current study (16 samples for each group) was greater than what was reported in most of previous studies [20]. Consequently, 80 premolars with 160 root-canals were sectioned 2 mm above the cementoenamel junction. The root-canals were instrumented up to a size #35 by the 2-Shape rotary system (MicroMega, France). They were irrigated between instruments with 2 ml of 5.25% sodium hypochlorite (NaOCl). Upon instrumentation, root-canals were irrigated with 1 ml of 5.25% NaOCl which was activated sonically by the EndoActivator system (DentsplySirona, Switzerland) for 30 s. Then, each root-canal was irrigated with 1 ml saline, followed by 1 ml of 17% EDTA solution which was activated sonically for 30se. The residual irrigant was removed with a 1 ml distilled water, then the root-canals were dried with paper points. They were filled with BioRoot RCS (Septodent, France) and gutta-percha (GP) cones (Meta Biomed, Republic of Korea) using the modified hot technique. The sealer was mixed according to the manufacturer instructions and then was inserted into the root-canals by the gutta percha master cone, during which efforts were made to standardize the amount of sealer inserted to all root-canals. The modified hot technique was performed using the Buchanan System-B Pluggers and the Element-Free obturation system (Kerr Cor, CA, USA) (the heat source was adjusted at 120 °C). The penetration depth of the heat plugger was limited to 6 mm shorter of the working length and was reached within 3 s instead of the traditional 5 s associated with the continuous waves’ compaction technique. The quality of the root-canals fillings were radiographically checked by mesiodistal and buccolingual radiographs in a standard projection. After inserting a coronal temporary restoration, samples were re-examined under microscopic magnification to check the integrity of the roots (no cracks) and then they were incubated for six weeks at 37 ± 1 °C and 100% relative humidity.

### Groups’ sampling

The study included 10 groups according to the reciprocating or rotary systems to be used with or without PUI. Therefore, these groups were named and numbered from 1 to 10. Using the Google Random Number Generator (www.google.com), samples were randomly allocated to the 10 equal groups (16 samples each). Each sample was given a number from 1 to 80, then each number generated by the Google Random Number was allocated to the groups from 1 to 10, consecutively. Sampling was performed by one of the research team, not the operator. Randomization was confirmed statistically (there were no significant differences among the groups regarding the volume of the initial root-canals fillings; the mean volume was 6.03mm^3^) (*p* > *0.05*).

### First micro-CT scanning, reconstruction and 3-D analysis of the root-canals fillings’ volume

Samples were placed in the specimen chamber of the SkyScan-1173 high energy Micro-CT machine (BrukerSkyScan, Belgium). The positions and orientations of samples within the specimen’s chamber were marked. A flat-field correction was performed before scanning procedures to correct variations in the camera pixel sensitivity. After adjusting the appropriate scanning parameters (image pixel size of 36.8 µm, exposure time of 675 ms, brass 0.5 mm filter, 0.4 rotation-step for 360° angle, 4 frame-average, and random movement of 8 to minimize ring artefacts), samples underwent the first scanning. Using the ©N-Recon (1.6.9.4) software (BrukerSkyScan), the projected images were reconstructed to produce 2-Dimensional (2D) cross-sectional images of the inner structure of the samples. A 5 ring artifact reduction for non-uniformity of the background image taken by the X-ray camera, 25% beam hardening compensation to prevent the specimen from appearing artificially denser at or near its surface and less dense at its central parts, and a smoothing of 2 using Gaussian kernel were applied. The reconstructed images were loaded to the Data-viewer software (1.5.6.2) (BrukerSkyScan) to determine images’ quality, reorient, resize, accurate positioning and visual inspection. A registration data-set was saved and loaded in the ©CTAn software (1.20.8.0) (BrukerSkyScan) to binarize and analyze images selectively to measure the volume of the root-canal filling material (mm^3^).

### Removal of the fillings’ materials (retreatment procedures)

The study’s groups were as follows:

- *WOG-Group* in which the fillings’ materials were removed using the #35 *WaveOne-Gold* files (DentsplySirona) powered by the Silver-Reciproc motor (VDW, Germany).
- *RB-Group* the fillings’ materials were removed by the *Reciproc-Blue* R40 file (VDW) powered by the Silver-Reciproc motor (VDW).
- *RM-Group* the #40 *R-Motion* reciprocating files (FKG) were used by the Silver-Reciproc motor (VDW) adopting the same parameters of the *RB* and WOG systems.
- *FAFO and TE Groups* for the *Fanta-AF-One* (#35/06) (Fanta Dental) and the *Tango-Endo* (#30/04) (Essential Dental Systems) *rotary* files rotated at 400 rpm rotation speed and 3.5 N/cm^2^ torque.
  Each *reciprocating* and *rotary* file was used for removal of the filling materials from three canals. Filling materials’ removal was deemed when the working-length of each root-canal was reached with the file five times and no filling material was found on the file [26]. The time to complete the retreatment procedures and any complication such as ledge formation or files separation, if any, were recorded.
- *In the  WOG-US, RB-US, RM-US, FAFO-US and the TE-US* groups, the filling materials were removed by the same *rotary* and *reciprocating* systems as in the above-mentioned groups, respectively, then additionally with PUI. Root-canals were filled with 5.25% NaOCl then the Silver-Tip (#20) (Eighteeth Medical Technology, China) was inserted 2–3 mm shorter of the working-length and ultrasonically activated for 20 s by the Ultra-x ultrasonic handpiece (Eighteeth Medical Technology) under 3.5X loups magnification. The PUI cycle was performed three times. The residual irrigant was removed with a final rinse of 2 ml distilled water. Each Silver-Tip was used in 3 canals.

### The second micro-CT scanning, reconstruction and 3-D analysis of the RFMs

**Fig. 1:**
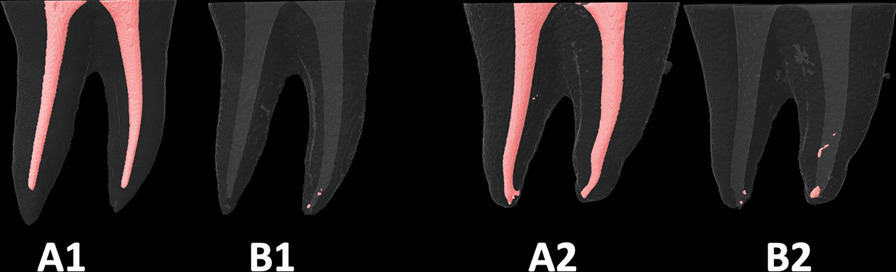
The root canal fillings **A1** and **A2** were removed using the *WaveOne-Gold* reciprocating system only (**B1**) or with suplimentary use of Passive Ultrasonic Irrigation (**B2**)

**Fig. 2:**
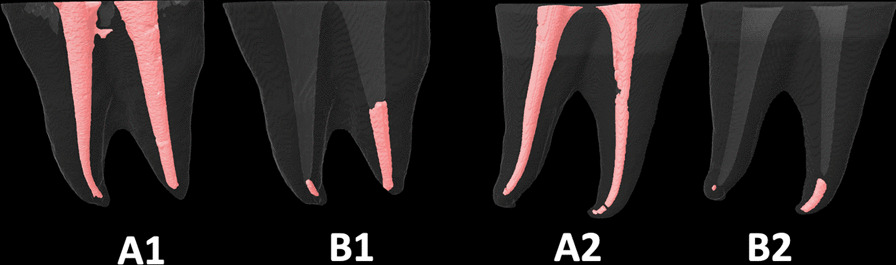
The root canal fillings **A1** and **A2** were removed using the *Fanta-AF-One* rotary system only (**B1**) or with suplimentary use of PUI (**B2**)

**Fig. 3:**
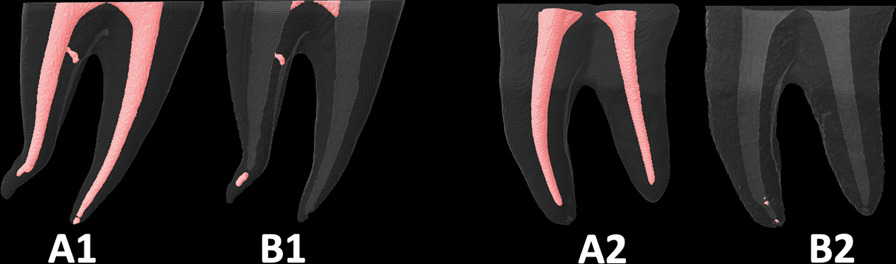
The root canal fillings **A1** and **A2** were removed using the *Reciproc Blue* reciprocating system only (**B1**) or with suplimentary use of PUI (**B2**)

**Fig. 4:**
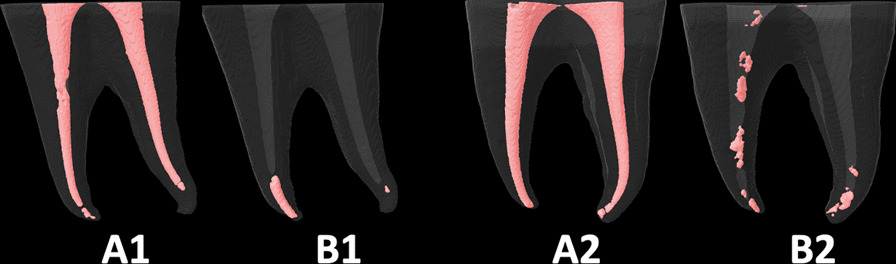
The root canal fillings **A1** and **A2** were removed using the *Tango-Endo* rotary system only (**B1**) or with suplimentary use of PUI (**B2**)

**Fig. 5:**
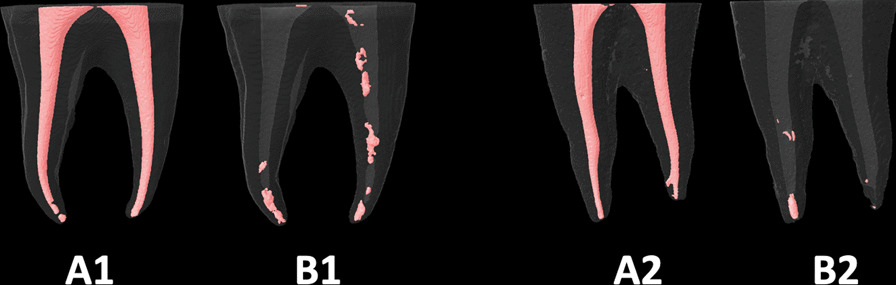
The root canal fillings **A1** and **A2** were removed using the *R-Motion* reciprocating system only (**B1**) or with suplimentary use of PUI (**B2**)

Samples were mounted within the micro-CT machine’s chamber in the same positions and orientation as in the first Micro-Ct scanning. Also, the Data-viewer software (1.5.6.2) (BrukerSkyScan) helped to maximize this repositioning of samples. Then they were scanned using the same scanning parameters as in the first scan. Also, the reconstruction (using the NRecon software) and the 3-D analysis (measurement of the volume of the RFMs) were carried out the same way as in the first 3-D analysis of the initial filling materials (before retreatment procedures). Figures 1, 2, 3, 4 and 5, represent samples of study groups before and after retreatment procedures; with and without passive ultrasonic irrigation.

### Calculation and statistical analysis

The percentage of the RFMs volume was calculated using the following equation [27]:

$$\frac{Volume\;of\;RFMs}{{Volume\;of\;original\;filling\;material}} \times 100 = Volume\;\left( \% \right)\;of\;RFMs$$

Data were entered into SPSS software version 20 (SPSS Inc, IL, USA) to calculate the mean of the RFM’s volume percentage for each group. The Sapiro-wilk normality test showed normal distribution of data (*p* > *0.05*). Therefore, Independent Samples-T, one-way ANOVA and two-way ANOVA statistical tests were used at *0.05* significance.

## Results

Overall, the RFMs after using *rotary* systems (10.1%) was significantly greater than that after using *reciprocating* systems (3.8%) (*P* < *0.001*) (Table 1). The mean RFMs percentage after using *WOG* and *RB* systems (2 and 2.6%, respectively) were significantly less than those recorded in the *RM*, *TE* and *FAFO* systems (6.8, 9.5 and 10.7%, respectively) *[P* < *0.05].* The *FAFO* system resulted in the significantly highest RFMs mean (10.7%) (*P* < *0.001*) but not significantly different from that of the *TE* system (9.5%) *[P* = *1.00]*.

**Table 1:** Mean and Standard Deviation of the ROMs’ volume (%) and Time required for attempt at removing the obturation materials (min) with or without PUI ultrasonics vibration (US)

	Retreatments without PUI					Retreatments with PUI				
	Reciprocating systems			Rotary systems		Reciprocating systems			Rotary systems	
	WOG (16)	RB (16)	RM (16)	FAFO (16)	TE (16)	WOG-US (16)	RB-US (16)	RM-US (16)	FAFO-US (16)	TE-US (16)
ROM volume (%)	2 ± 0.7^a^	2.62 ± 1.1^b^	6.8 ± 4.3^a, b, c^	10.70 ± 4.1^a, b, c, d^	9.51 ± 3^a, b, c^	1.48 ± 1.2	1.31 ± 0.9	1.16 ± 0.9	1.88 ± 0.9	1.4 ± 1.1
Total	(48) 3.8 ± 3.32			(32) 10.13 ± 3.57		(48) 1.32 ±			(32) 1.64 ± 1.05	
	(No: 80) *P* < 0.001					(No: 80) *P* = 0.225				
	*6.27* ± 4.6					1.45 ± 1.0				
	*P* < 0.001									
Removal time (min)	4.9 ± 0.7^a^	5.4 ± 0.6^b^	4.1 ± 0.6^a, b, c^	4.1 ± 0.5^a, b, d^	3.7 ± 0.5^a, b, e^					
Total	4.8 ± 0.8			3.9 ± 0.5						
	*P* < 0.001									

Symmetrical letters indicate a significant different between paired groups (*p* < *0.05*)

The mean times required to remove the root-canals’ filling materials using the *TE, FAFO* and *RM* systems (3.7 and 4.1 min, respectively) were significantly lower than those required by the *RB* and *WOG* rotary systems (5.4 and 4.9 min, respectively) *[P* < *0.05]*. Overall, the time required to remove the filling materials using the *rotary* systems (3.9 min) was significantly less than that required by the *reciprocating* ones (4.8 min) [*P* < *0.001*].

Overall, using supplementary PUI resulted in a significantly less RFMs (1.44%) when compared to using only *rotary* or *reciprocating* systems without PUI (6.27%) *[P* < *0.001]*. This was applied to all groups (*P* < *0.05*) except the *WOG* one (*P* = *0.65*).

## Discussion

Retreatability is one main criterium of root-canals filling materials. CSCSs were developed and introduced to improve the long-term outcomes. However, their retreatability was one main concern. The literature has shown inconsistent results in this regard. Some studies found that retreatability of CSCSs and AH-Plus sealers is comparable [5, 28]. While Uzunoglu et al. [6] observed greater RFMs with MTA-based sealer compared to AH-26, others reported less RFMs when MTA-types sealer was compared to that of AH-Plus sealer [9, 29]. The differences in assessment methods, storage times for sealers setting, adhesion to dentine properties, obturations’ extension and techniques and anatomies of root-canals may explain these conflicting results. All root-canals (100%) in the current study deemed patent after retreatments. Also, the current study’s scope was not to compare different CSBSs, rather than to investigate the retreatment ability of different engine-driven files. Therefore, the BioRoot RCS (Septodent), which is a tricalcium silicate-based sealer, was used as an example of CSCSs with gutta-percha to fill the root-canals in the current study. Nevertheless, the current study results were comparable to those obtained in previous studies and confirmed the retreatability of these types of sealers.

Different methods have been implemented to assess the retreatment ability of endodontic instruments. Two-D assessment, which includes radiographic analysis [30], scanning electron microscopy [31], tooth clearing [32] and analysis of split teeth images [33], has some drawbacks. The 2-D radiographs method, for example, provides only 2-D information and may show some distortions of the 3-D structures and cannot visualize small volumes of debris [30]. The vertical split-teeth method is destructive and provides only 2-D measurement of the RFMs [19]. Also, some of the RFMs can be lost during the vertical splitting procedures. Three-D measurements by cone-beam computed tomography (CBCT) or micro-CT are more accepted tools as they are non-destructive and enable more accurate 3-D measurements with different interventions [23, 34]. Nevertheless, the only disadvantages of micro-CT assessment are cost and time-consumption.

No solvents were used in this study, which might be a limitation. However, their role in enhancing removal of filling materials is still debatable [35–37]. Some researchers recommend using solvents whenever the working length can not be reached as they reduce the time to reach the working-length, though don’t improve root-canal cleanliness and may cause blockage of dentinal tubules by gutta-percha and sealers [35, 38, 39]. Although solvents’ extrusion is below the permissible toxic dose, hence risk to patients can be negligible [40], future studies are needed to confirm their long-term safety.

Some researchers claim that removal of teeth’ crowns may result in better standardization of the working-length and filling-materials’ removal and eases accessing the root-canal system [33, 41]. However, the impact of the limited access to root-canals on the instruments’ retreatment ability should not be overlooked, because clinically teeth undergoing retreatments have crowns. Also, even with keeping the crowns, standardization can still be achieved by standard working-lengths and standard access cavities. Teeth in the current study were sectioned 2 mm above the cementoenamel junction. This reflects the clinical situations, to extent, in which retreatment usually is performed on heavily destructed teeth. Also, it might be argued that roots were better scanned before instrumentation to confirm standardization, hence it maybe considered as another study’ limitation. Such step is essential when investigating aspects of instrumentation, i.e. shaping ability of different instruments. However, the objective of the current study was to investigate the changes of the volume of the root canal filling after retreatment procedures, hence it is important to standardize volumes of the root-canals fillings. There were no significant differences among the study groups regarding the volume of the initial root-canals filling materials, which is due to the fact that all root-canals were instrumented to the same apical size preparation, and randomly were allocated to different groups.

In accordance with previous studies, all samples, in this study, exhibited some RFMs [17, 23]). It is well accepted that no endodontic file can instrument the entire root-canals walls [42]. However, implementing advanced tools may result in less RFMs [23, 33]. Supplementary agitation of irrigants by sonic or ultrasonic vibrations or laser irradiation resulted in significantly less RFMs [14–17]. The current study also showed that PUI after using rotary or reciprocating systems resulted in significantly less RFMs. This was applied to all groups except the *WOG* group which exhibited insignificant reduction of the RFMs (Fig. 1). The reason could be that the *WOG* group had the least RFMs in the first instance. The acoustic streaming within the irrigant solution, generated by the oscillating tip, produces hydrodynamic action that may enhance removal of the filling-materials, especially that impeded in root-canals areas which cannot be reached by files. Streaming depends upon the operating conditions; i.e. power-settings, constrains, composition and dimensions of ultrasonic tips, and pressure applied on them [43]. Nevertheless, some studies found no improvement in removal of the filling materials upon irrigants agitation [44, 45]. This inconsistency maybe due to different root-canal filling-materials, different retreatment procedures and different root-canals anatomies.

The literature also shows inconsistent effectiveness of *reciprocating* and *rotary* systems [23] which can be explained again by the different research’ methodologies (using plastic blocks or extracted teeth, curvatures’ angles and shapes of the root-canals, types and quality of initial root-canals filling materials, supplementary retreatment procedures, sizes of instruments, and assessment’ methods). Some studies revealed that *reciprocating* systems were as effective as *retreatment rotary* ones [9, 18–21]. However, the *reciprocating* systems in our study were more effective in removing the filling materials than *rotary* systems. These findings in agreement with those obtained in some previous studies [18, 26, 46]. Unlike De-Deus et al. [47, 48] some authors indicated that larger sizes instruments can remove more root-canals filling materials. However, this study showed that instruments sizes and tapers did not influence their effectiveness. The *WOG* was better than the *FAFO* (Fig. 2), though both had a 35 ISO tips’ size. Also, the *RB* (size 40) (Fig. 3) was as effective as the *WOG* (size 35). Though the *FAFO* and the *TE* (Fig. 4) had different tapes (06 and 04, respectively) they resulted in statistically similar RFMs volume means. Madarati et al.explained that the reciprocating motion was the main reason for the good effectiveness of the *Sendoline-1*, *Reciproc* and *WaveOne* files, despite their different tapers, sizes, and cross-section shapes [19]. The alternating motion of the reciprocating files could better dislodge the filling material, especially the hard set MTA-sealer from the root-canal walls, enhancing its removal coronally if the instrument design (cross-sectional shape and the helical angle) allowed such removal. Also, the reciprocating systems have better centering ability than rotary systems, which could be an additional reason [33, 49]. All reciprocating files, in this study, were rotated at 150° counterclockwise/50° clockwise angles, which neglects the influence of the reciprocating angles on instruments retreatment ability. However, the effect of the instruments’ designs on their retreatment ability cannot be neglected and could be the reason for the inferior effectiveness of *RM* (Fig. 5) compared to *WOG* and *RB* systems. One possible reason is the different number of contacts of the cutting-edges with canals’ walls. Though the *WOG* has a parallelogram cross-section shape with four cutting-edges, it has 1 or 2 alternating cutting-edges contact with canals’ walls depending on the location along the file (https://www.dentsplysirona.com/en). Also, the S-shape cross-section of the *RB* allows two contact edges. By contrast, the *RM*, with a rounded-triangular symmetrical cross-section, has three contact cutting-edges. It is important to differentiate between the number of contacts of cutting-edges with canals walls and the sum of contact area. Both of *FAFO* and *TE* files have completely flat side along their active part. Both files’ designs result in a greater space for the accumulation of the removed filling materials inside files’ flutes compared to that with *reciprocating* files (*WOG, RB* and *RM*). This may be another factor, in addition to the different motions’ modes, that explains the inferiority of rotary systems (*FAFO* and *TE*) compared to the reciprocating ones. Nevertheless, further investigations of the impact of different rotation motion’s modes (reciprocating, continuous or adaptive motion) using the same files design is paramount.

Overall, the time required to remove the root-canals’ filling materials by *rotary* systems was significantly shorter than that required by the *reciprocating* ones; with the shortest times recorded by the *TE* and *FAFO* systems (3.7 and 4.1 min, respectively). The operator noticed that the latter two systems were faster during the initial penetration of the root canals’ fillings than the *RB* and *WOG* files. However, easier penetration of instruments into the root-canals’ fillings does not imply greater removal. Also, our results showed no correlation between instruments’ retreatment ability and the time they required to perform retreatment procedures. These findings were consistent with those reported in a previous study [6]. It is believed that using fewer number of files can result in shorter retreatment procedures. However, the literature shows inconsistency in this regard. While some studies reported no significant difference between *rotary retreatment* (designed specifically for retreatment) and *reciprocating* files [19, 50, 51], others found that the *retreatment* rotary files were faster than *reciprocating* files [52]. By contrast, Zuolo et al. [53] found that the *Reciproc* system was faster than the *Mtwo-R* retreatment files. Again, different research methodologies can explain these different findings. Nevertheless, the time needed for removing root-canals filling materials should not be overvalued as an essential factor for choosing retreatment instruments rather than being correlated with the cleaning effectiveness during retreatment.

## Conclusions

Within the conditions of the current study, the following can be concluded:

- The *reciprocation* systems (*WOG*, *RB* and *RM*) were more effective in removing CSCSs fillings than *rotary* systems (*TE* and *AFOF*)
- The PUI, as a supplementary procedure, improved removal of the root-canals’ filling materials.
- The ability of *rotary* and *reciprocating* systems to remove filling materials was not correlated to the time they required for removal attempts.
- The rotary systems (FAFO and TE) needed shorter times for removal of fillings material compared to *reciprocating* systems (*WOG*, *RB* and *RM*).

## Data Availability

The data that support the findings of this study are available from the National Science Technology and Innovation Plan (KSA) (https://npst.ksu.edu.sa/en) but restrictions apply to the availability of these data, which were used under license for the current study, and so are not publicly available. Data are however available from the authors upon reasonable request and with permission of the National Science Technology and Innovation Plan (KSA).

## Abbreviations

RCTs: Root-canals treatments
CSCSs: Calcium silicate-containing sealers
RFMs: Remaining fillings materials
CT: Computed tomography
CBCT: Cone-beam computed tomography
3-D: Three dimensional
2-D: 2-Dimensional
WOG: WaveOne-Gold
RB: Reciproc-Blue
RM: R-Motion
FAFO: Fanta-AF-One
TE: Tango-Endo
PUI: Passive ultrasonic irrigation
